# A project of harmonious health renewal in psychoneurological patients on the basis of the personalized algorithms for treatment and rehabilitation

**DOI:** 10.1186/1878-5085-5-S1-A95

**Published:** 2014-02-11

**Authors:** Ulyana B Lushchyk, Viktor V Novytskyy, Igor P Babii, Nadiya G Lushchyk, Olena V Babyuk, Valentina V Leonova, Ivanna I Legka

**Affiliations:** 1 Research Center Veritas, 31 Obolonska Str., of. 9, Kyiv, 04071, Ukraine; 2 The Clinic of Healthy Vessels, 4 Williams Str., Kyiv, 03191, Ukraine; 3 Center for Innovative Medical Technologies Veritas IT Med, 4 Williams Str., Kyiv, 03191, Ukraine

## Scientific objectives

Problems of psychoneurological patients’ treatment always were and remain actual one as the nervous system’s structure is complicated and treatment results are sometimes unpredictable [1]. Today new knowledge and intellectual technologies enable to reconsider generally accepted approaches.

## Results interpretation

The Veritas Research Center has made the comparative analysis of standard and multidisciplinary approaches to treatment of 724 psychoneurological patients, who were treated and rehabilitated in the Clinic of Healthy Vessels. 78% had psychosomatic disorders, 63% of cases required psychotherapy, 47% - psychocorrection, 38% - psycho-speech correction, 83% - motion disorders. 23% of patients had lasting patterns of psychological guidelines "on illness", aggravation, adjusting behaviour on the disease progress and block of the future vision. For the first month of treatment in the result of correction of the educed concomitant pathologies and rehabilitation the treatment effect has grown on 15-21% depending on the expressed psychoneurological lack. During the long-term rehabilitation for 3 months up to 1 year the treatment costs have reduced on 10-17% in comparing to standard approach and the treatment effect has grew on 75-83% [2,3]. For a year of the individually oriented multidisciplinary rehabilitation and treatment 84% of serious non-curable patients with apallic syndrome, ICP, epilepsy has reached 5^th^ score of the Rancho Los Amigos Scale from the lower level [2,3].

## Outlook and expert recommendations

According to the results of the conducted researches it is expedient to change the treatment algorithms for psychoneurological patients with the purpose of more rational use of costs and reaching greater effect, bringing the nervous system on the autoregulation level and self-control of neuronets, renewal of self-service and capacity.

## References

[B1] Lushchyk UB Novytskyy VV Polyvector dynamic diagnostics of the vascular systems. A modern innovative medical technology: from local examinations to integrative comprehension of the whole system Med Devices 2008 3 64 67

[B2] Lushchyk UB Babii IP Titenko TM Novytskyy VV Stukalin VO Lushchyk NG Leonova VV Priz AM Innovative vectors in neurorehabilitation. Logic and management of multidisciplinary approach in restorative medicine Macros 2012 244 Kyiv

[B3] Lushchyk UB Novytskyy VV Alexeyeva TS Francevich KA Branytska NS Analytical aspects of an individual hemodynamic correction in the angioneurology Istyna 2006 130 Kyiv

